# Recurrent mutations, including NPM1c, activate a BRD4-dependent core transcriptional program in acute myeloid leukemia

**DOI:** 10.1038/leu.2013.338

**Published:** 2013-12-13

**Authors:** M A Dawson, E J Gudgin, S J Horton, G Giotopoulos, E Meduri, S Robson, E Cannizzaro, H Osaki, M Wiese, S Putwain, C Y Fong, C Grove, J Craig, A Dittmann, D Lugo, P Jeffrey, G Drewes, K Lee, L Bullinger, R K Prinjha, T Kouzarides, G S Vassiliou, B J P Huntly

**Affiliations:** 1 Department of Haematology, Cambridge Institute for Medical Research and Addenbrookes Hospital, University of Cambridge, Cambridge, UK; 2 Wellcome Trust—Medical Research Council Cambridge Stem Cell Institute, Cambridge, UK; 3 Gurdon Institute and Department of Pathology, University of Cambridge, Cambridge UK; 4 Haematological Cancer Genetics, Wellcome Trust Sanger Institute, Hinxton, UK; 5 Discovery Research, Cellzome AG, Heidelberg, Germany; 6 Epinova DPU, Immuno-Inflammation Centre of Excellence for Drug Discovery, GlaxoSmithKline, Medicines Research Centre, Stevenage, UK; 7 Department of Internal Medicine III, University Hospital of Ulm, Ulm, Germany

**Keywords:** acute myeloid leukemia, epigenetic therapy, BET protein, nucleophosmin mutation, biomarker

## Abstract

Recent evidence suggests that inhibition of bromodomain and extra-terminal (BET) epigenetic readers may have clinical utility against acute myeloid leukemia (AML). Here we validate this hypothesis, demonstrating the efficacy of the BET inhibitor I-BET151 across a variety of AML subtypes driven by disparate mutations. We demonstrate that a common ‘core' transcriptional program, which is *HOX* gene independent, is downregulated in AML and underlies sensitivity to I-BET treatment. This program is enriched for genes that contain ‘super-enhancers', recently described regulatory elements postulated to control key oncogenic driver genes. Moreover, our program can independently classify AML patients into distinct cytogenetic and molecular subgroups, suggesting that it contains biomarkers of sensitivity and response. We focus AML with mutations of the Nucleophosmin gene (*NPM1*) and show evidence to suggest that wild-type NPM1 has an inhibitory influence on BRD4 that is relieved upon NPM1c mutation and cytosplasmic dislocation. This leads to the upregulation of the core transcriptional program facilitating leukemia development. This program is abrogated by I-BET therapy and by nuclear restoration of NPM1. Finally, we demonstrate the efficacy of I-BET151 in a unique murine model and in primary patient samples of NPM1c AML. Taken together, our data support the use of BET inhibitors in clinical trials in AML.

## Introduction

Acute myeloid leukemia (AML) is an aggressive hematological malignancy where <30% of all patients are long-term survivors.^1^ Over 11 000 patients per year die of this disorder in the United States alone and novel therapeutics are urgently required. Although AML is highly heterogeneous at the genetic and biological level, unifying themes are evident.^2, 3, 4^ These include aberrant transcription and epigenetic dysfunction, which provide a potential basis for therapeutic intervention.^5^ In particular, the dynamic plasticity of the epigenome lends itself well to therapeutic manipulation. As an exemplar of this principle, we and others have recently shown the efficacy and mechanism of action of protein–protein interaction inhibitors of the bromodomain and extra-terminal (BET) family of epigenetic readers in animal models of mixed lineage leukemia (MLL)-rearranged leukemias,^6, 7^ multiple myeloma^8^ and non-Hodgkins lymphoma.^9^ However, the efficacy and potential mechanism(s) of action of BET inhibitors in other forms of AML are largely unknown.

One of the most common mutations in AML, occurring in 35% of cases, involves the nucleophosmin (*NPM1*) gene.^10^ NPM1 is a pleiotropic protein with roles in processes as diverse as ribosome biogenesis, chaperoning histones and centrosome duplication. The functional integrity of NPM1 is dependent on its ability to shuttle between the nucleus and cytoplasm, and this ability is severely compromised in NPM1 mutated AML.^11^ The mutations (termed NPM1c mutations) uniformly alter one or both critical tryptophan residues in the C-terminus of the protein, which prevent proper folding^12^ and destroys a nucleolar localization signal. In addition, the most common mutation (type A, accounting for 75% of all mutations)^11^ also generates an aberrant extra nuclear export signal. Although NPM1c mutations are heterozygous, hetro/homodimerization with wild-type (WT) NPM1 results in cytoplasmic mislocalization of both mutant and WT protein. This alteration in subcellular location perturbs normal NPM1 function, including the mislocalization and stabilization of critical proteins such as the TP53 regulator p14ARF,^13, 14^ and leads to transformation. This process also generates a distinct transcriptional signature in NPM1c AML that facilitates the generation of leukemia.^15, 16^ However, the nature of aberrant transcriptional regulation in NPM1c AML remains obscure.

In this report, we address these questions and demonstrate that multiple AML subtypes are sensitive to BET inhibition *in vitro*. In addition, we identify a BRD4-dependent transcriptional program that maintains multiple subtypes of AML and underlies I-BET sensitivity. In mechanistically interrogating the sensitivity of the common NPM1c subtype of AML, we further identify a previously unknown function for NPM1 as a negative regulator of BRD4-dependent transcription. This function is perturbed in NPM1c AML and inhibited by I-BET. Finally, we demonstrate convincing preclinical evidence to suggest efficacy of BET inhibition in NPM1c AML.

## Materials and methods

### Cell culture

MV4;11, MOLM13, NOMO1, Kasumi, ME-1, SKM1, KG-1, NB4, HEL, HL60 and K562 were grown in RPMI-1640 medium (Sigma-Aldrich, St Louis, MO, USA) supplemented with 10–15% fetal calf serum (FCS). OCI-AML3 were grown in 80% alpha-MEM+20% FCS. All growth media were 1% penicillin/streptomycin. Murine progenitors retrovirally transformed with MOZ-TIF2 or NUP98-HOXA9 were grown in RPMI-1640 medium supplemented with 20% FCS+10 ng/ml of IL3. Primary human leukemia cells were also grown in RPMI-1640 medium supplemented with 20% FCS in the presence of 10 ng/ml of IL3, 10 ng/ml IL6 and 50 ng/ml SCF. Cells were incubated at 37 °C and 5% CO_2_. K562 cells were transfected using the Amaxa Nucleofector system according to the manufacturer's instructions. Cell proliferation assays and clonogenic assays in methylcellulose were performed as previously described.^6^ Immortalized MOZ-TIF2 and NUP98-HOXA9 cell lines were generated through retroviral transduction of whole bone marrow with MSCV MOZ-TIF2 and MSCV NUP98-HOXA9, as previously described.^26^

### Gene expression and chromatin immunoprecipitation

Gene expression analysis and chromatin immunoprecipitation followed by downstream analysis with next-generation sequencing or RT-PCR were performed as previously described.^27^ The following primer pairs were used in the chromatin immunoprecipitation analysis.


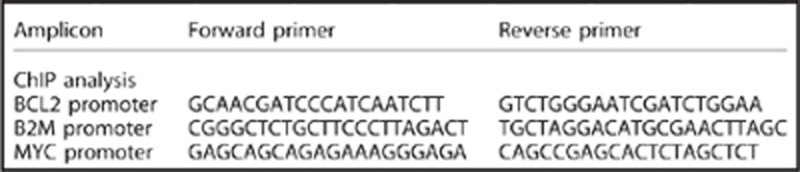


The following primer pairs were then used in the gene expression analysis.


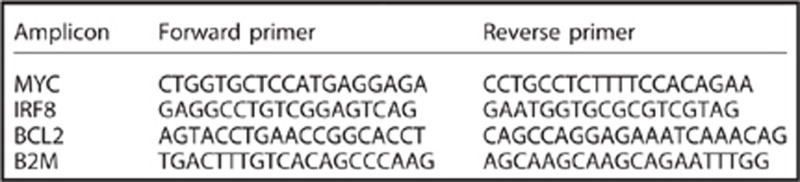


### Murine models of disseminated NPM1c leukemia

Three separate NPM1c AML samples each containing different collaborating mutations were chosen for *in vivo* studies. Ten million NPM1c AML cells from each leukemia were intravenously injected into 6–8-week-old sublethally irradiated (300 cGy) NOD-SCID mice, as previously described.^6^ Treatment with I-BET at 15 mg/kg was commenced on day 11 and mice were inspected twice daily. This dosing schedule maintained plasma levels of the compound above the *in vitro* IC_50_ (Supplementary Figure 1). Mice were killed upon signs of distress/disease. All mice were kept in a pathogen free animal facility. All experiments were conducted under UK home office regulations. Mouse histology and tissue sample preparation were performed as previously described^6^

### Flow cytometry analysis

Cell apoptosis and cell cycle analysis were performed as previously described^6^ on an ADP flow cytometer (Dako, Stockport, UK), and all data analyzed with FlowJo software (Tree Star, Inc., Ashland, OR, USA).

### Patient material

Peripheral blood or bone marrow containing >80% blasts, was obtained from patients following consent and under full ethical approval at each involved institute.

### Antibodies

The following antibodies were used in ChIP assays, western blotting assays and for immunoprecipitation: anti-H3 (ab1791; Abcam, Cambridge, UK), anti-BRD4 (A301-985A; Bethyl Labs, Montgomery, TX, USA); anti-GFP (ab290; Abcam) and Rabbit polyclonal IgG (ab27472; Abcam). Antibodies used for immunofluorescence were anti-BRD4 (ab75898; Abcam) and Alex Fluor-488-conjugated IgG (Invitrogen, Paisley, UK) immunofluorescence 1:250.

#### Immunofluorescence microscopy

Haematopoietic cells were washed once in 1 × phosphate buffered saline before cytocentrifugation onto polylysine coated microscope slides. Cells were fixed with buffered 4% paraformaldehyde, and following stepwise incubation with primary and then secondary fluorescent antibody (see antibodies) cells were stained with Hoechst 33258 (Sigma-Aldrich) and mounted with Vectashield mounting medium (Vector Laboratories, Peterborough, UK). Confocal laser images were captured with an Olympus Fluoview FV1000 microscope equipped with a 40 × oil lens (Olympus, Southend-on-Sea, UK). Image processing was carried out using PHOTOSHOP (Adobe systems, San Jose, CA, USA).

### Bioinformatics analysis

#### Microarray and bioinformatics analysis

RNA from OCI-AML3 was extracted after 6 h of treatment with I-BET151 and processed as described^6^ before hybridization to Illumina Human HT12 v4 BeadChips (Illumina, San Diego, CA, USA). Gene expression data were processed using the lumi^28^ package in R. Probes were filtered to remove those where the detection *P*-value (representing the confidence that expression is above the background of the negative control probe) was greater than 0.01 in all samples. Expression data were transformed using variance stabilization,^29^ then normalized using quantile normalization. Comparisons between the dimethyl sulfoxide- and BET-inhibitor-treated samples for all three cell lines (OCI-AML3, MV4;11 and MOLM13) were performed using the R package limma. Genes with a false discovery rate below 5% and a fold-change greater than two were considered significant. Agglomerative hierarchical clustering of the most downregulated genes was performed in R using complete linkage on the pairwaise Euclidean distance between gene signatures, and clusters were identified based on a cutoff of 10.

#### ChIP-seq analysis

Sequenced reads were mapped to the reference human genome (hg19) using the Burrows–Wheeler aligner^30^ with default parameters. Only reads mapped with a mapping quality score >10 were retained, and multiple reads mapping to identical genomic loci were removed to limit potential PCR bias. Reads were extended along their strand to the estimated fragment size of 300 bp. Peaks were called using Model-based analysis of ChIP-seq^31^ with standard parameters. The distribution of reads about the transcriptional start site of all protein-coding genes >1 kb, as well as lincRNAs, miRNAs, snoRNAs and snRNAs, was calculated using the Repitools package in R.

#### Assigning enhancer and super-enhancer regions to specific genes

Enhancer and super-enhancer regions were defined as per Young and colleagues, based upon ChIP-seq enrichment for the mediator complex member MED1, the histone mark H3K27Ac and distance from promoters as defined by H3K4Me3 in the MMI.S cell line.^17^ As there was an exceptionally strong correlation between MED1 and BRD4 binding intensity at enhancer regions in MM1.S cells, we estimated the number of ChIP-seq reads mapped to these enhancer regions for BRD4 binding in the OCI-AML3 cell line following treatment with dimethyl sulfoxide and I-BET, respectively. For both the treatments, reads per million were estimated and enhancer regions were ranked according to increasing BRD4 signal. Genes (*N*=6725) that were within 50 kb were assigned to the enhancer regions. Only a few genes were assigned to more than one enhancer.

## Results

### BET inhibition demonstrates efficacy across a number of AML subtypes

**Figure 1:**
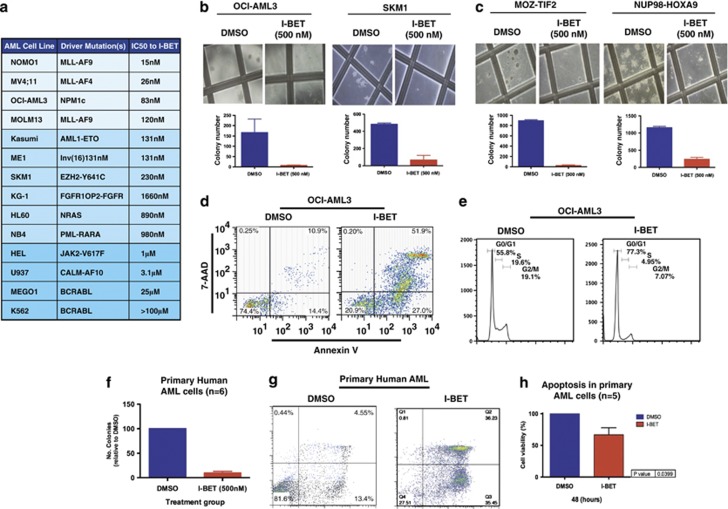
I-BET151 has activity in a broad range of AML (**a**) A panel of human AML cell lines encompassing a variety of oncogenic drivers were tested in cell proliferation assays using I-BET151. We have previously reported some of this data^6^ and report it again only to provide an overall appreciation of sensitivity of AML cell lines to I-BET151. (**b**) Clonogenic assays performed in cytokine-supplemented methylcellulose in the presence of vehicle (dimethyl sulfoxide (DMSO)) or I-BET151 show a marked reduction in colony numbers (enumerated in the bar graph) after treatment with I-BET151. (**c**) Primary murine hematopoietic progenitors were isolated from mouse bone marrow and retrovirally transformed with MOZ-TIF2 or NUP98-HOXA9. These cells were propagated in liquid culture as well as being used in clonogenic assays. Both proliferation and clonogenic assays (enumerated in the bar graph) demonstrate a marked sensitivity to I-BET151. (**d**) The degree of apoptosis in OCI-AML3 was assessed using the vital dye 7-amino-actinomycin D (7-AAD) and Annexin V in cells following 72 h incubation with DMSO or I-BET. These data demonstrate a marked induction of apoptosis. (**e**) Cell cycle progression in OCI-AML3 was assessed 24 h after incubation with DMSO or I-BET151. These data demonstrate a marked increase in G_0_/G_1_ fraction, which was accompanied by a concomitant decrease in the number of cells in S and G_2_/M phases. (**f**) Clonogenic assays with primary human AML cells from five different patients (Supplementary Table S2). Cells were plated in cytokine-supplemented methylcellulose in the presence of vehicle (DMSO) or I-BET151. These show a marked reduction of colony formation in the presence of I-BET151. AML patient samples demonstrate apoptosis following treatment with I-BET. A representative sample is shown (**g**) and the results from five separate patients are enumerated in the bar graph (**h**).

To determine if BET proteins are valid therapeutic targets in other AML subtypes, we assessed the sensitivity of a representative panel of cell lines with common recurrent AML mutations to inhibition by I-BET151 (hereafter I-BET) in growth and colony formation assays (Figures 1a and b and data not shown). These data demonstrate that in addition to cell lines harboring MLL translocations, a number of AML cell lines including OCI-AML3 (which is the only human cell line that contains an NPM1c mutation), KG-1 (FGFR1OP2-FGFR1 rearrangement), SKM1 (EZH2 Y641C mutation), Kasumi (AML1-ETO rearrangement) and ME-1 (CBFβ-MYH11 rearrangement) show sensitivity to treatment with I-BET, both in liquid culture and clonogenic assays (Figures 1a and b and data not shown). Moreover, murine bone marrow progenitors retrovirally transduced with poor-risk AML-associated fusion oncogenes for which no human cell lines exist, such as MOZ-TIF2 and NUP98-HOXA9 also demonstrated sensitivity (Figure 1c). Although the sensitivity varied over a relatively wide range, the majority of cell lines were inhibited at concentrations predicted to be achievable *in vivo* (Figure 1a and Supplementary Figure 1). Similar to our observations in the MLL-rearranged leukemias, I-BET induced a rapid and profound apoptosis and G_0_/G_1_ cell cycle arrest in non-MLL fusion AML cell lines (Figures 1d and e and data not shown). Finally, sensitivity to I-BET was also demonstrated in clonogenic assays in primary samples from patients with non-MLL fusion AML (Figure 1f and Supplementary Table 1), and I-BET was shown to induce apoptosis across multiple non-MLL fusion patient samples (Figures 1g and h). Taken together, our findings demonstrate the efficacy of I-BET against a broad range of AML cells with disparate oncogenic mutations and suggest clinical utility across a wide range of AML subtypes.

### BET proteins regulate a ‘core' transcriptional program in AML

**Figure 2:**
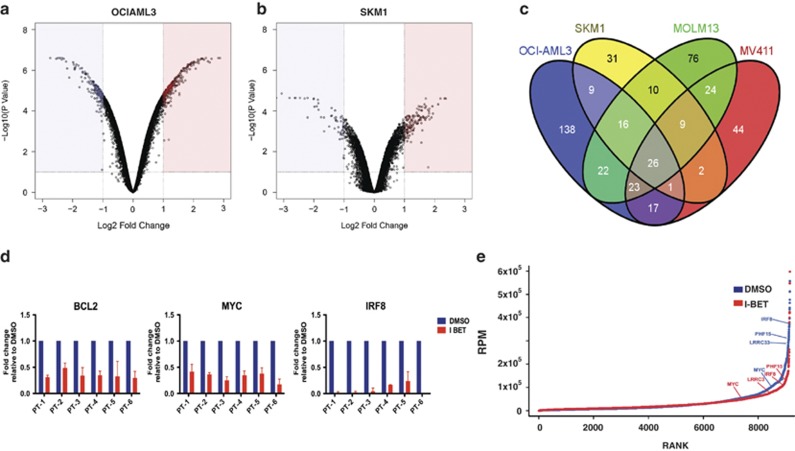
A core transcriptional program is affected by I-BET151 in AML. (**a**) OCI-AML3 and (**b**) SKM1 cells were treated for six hours with either I-BET151 or DMSO (vehicle) followed by mRNA extraction. The mRNA from three biological replicates was used to generate gene expression data set. Volcano plots for the DMSO- versus I-BET151-treated samples, showing the adjusted significance *P*-value (log10) versus the fold change (log2) are shown. These plots identify a small subset of genes that demonstrate a significant change in expression (*P*⩽0.01). This is represented as either twofold downregulation (blue) or twofold upregulation (red) on treatment with I-BET151. (**c**) Venn diagram of all the significantly downregulated genes, shows that 26 genes are commonly downregulated in all four cell lines. Several of these genes are also downregulated in another sensitive AML cell lines KG-1 (Supplementary Figure S3). (**d**) Similar transcriptional changes were demonstrated in both NPM1c mutated and wild-type AML patients, for exemplar, genes C-MYC, BCL2 and IRF8. (**e**) Total BRD4 ChIP-seq signal in units of reads per million is charted at all enhancer regions. Enhancers are ranked by increasing BRD4 ChIP-seq signal in the presence (red) or absence of I-BET151 (blue). Super-enhancers are enriched in the vertically rising ranked enhancers to the right of the graph. Treatment with I-BET151 markedly decreases the BRD4 read count at these enhancers.

We have previously demonstrated that inhibition of BET proteins alters a specific transcriptional program in MLL-rearranged leukemias, with downregulation of genes within this program, such as *BCL2* and *C-MYC*, leading to the induction of apoptosis and cell cycle arrest.^6^ On the basis of our demonstration of similar cellular phenotypic consequences following I-BET treatment in sensitive AML cell lines, we hypothesized that downregulation of a similar transcriptional program may have mediated these findings. To test this hypothesis, we analyzed the changes in gene expression following 6 h of I-BET treatment (before significant changes in cell cycle and apoptosis are evident) in the sensitive cell lines OCI-AML3 and SKM1, containing NPM1c and EZH2 Y641C mutations, respectively. Similarly to the MV4;11- and Molm13 MLL-rearranged cell lines, only a relatively small number of genes demonstrated a significant alteration in expression in either cell line, with the majority of genes unchanged (Figures 2a and b). This corroborates our previous finding of the specificity of inhibition of BET proteins on overall gene expression in AML. Although a number of genes were uniquely altered, remarkably, the degree of overlap of the transcriptional changes between OCI-AML3, SKM1 and each of the MLL cell lines was similar to the overlap between the two MLL cell lines themselves and there was a high degree of correlation between the genes (Figure 2c and Supplementary Figure 2A). Importantly, the expression of 26 genes was commonly downregulated in all four cell lines following I-BET treatment (Figure 2c and Supplementary Table 2). This gene set, which includes several critical regulators of myelopoiesis and leukemia including *BCL2, C-MYC* and *IRF8*, was also downregulated in other AML cell lines sensitive to I-BET (Supplementary Figure 2B). Moreover, decreased expression of a subset of the same genes was demonstrated in primary patient AML samples following I-BET treatment (Figure 2d and Supplementary Table 1). Of particular interest, genes from the *HOXA* cluster do not form part of this core transcriptional program (Supplementary Table 2). Taken together, this inhibition of common critical leukemia regulators following I-BET treatment in a range of AML subtypes demonstrates that BET proteins regulate the expression of a ‘core' transcriptional program in AML and that this program is *HOX* gene independent. Although BRD4 is present ubiquitously at promoter and enhancer elements, it has recently been demonstrated that the expression levels of certain genes are more susceptible to BRD4 inhibition.^17^ A feature of these responsive genes appears to be the presence of super-enhancers. Using the same methodology described by Young and colleagues,^17^ we find that nearly half (12/26) of the genes present within the core transcriptional program contain super-enhancers and BRD4 binding to these regions is dramatically decreased following just 6 h of inhibition with I-BET151 (Figure 2e).

### The BET-dependent core transcriptional program classifies patients with AML into groups that differ in their specific molecular subtype and may predict response to BET inhibition

**Figure 3:**
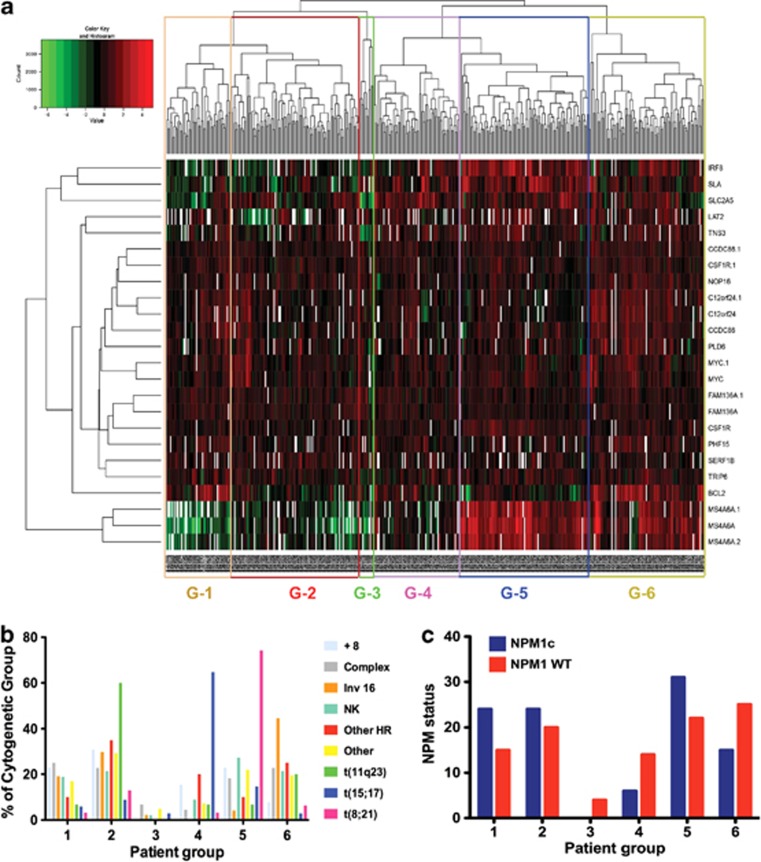
The core transcriptional program classifies human AML: (**a**) 18 of 26 genes (80%) from the BET-responsive core signature were differentially expressed across a cohort of 436 AML patients as shown in the heat map. The gene set could classify this cohort into six groups through the use of unsupervised clustering. (**b**) Significant differences in cytogenetic characteristics were shown for individuals in each of the groups (*P*<0.0001), with significant differences in molecular prognostic factors including mutational status for (**c**) NPM1c and FLT3-ITD (Supplementary Figure 3) also noted (*P*=0.02 and *P*=0.02, respectively). (+8=trisomy 8, NK=normal karyotype, Other HR=other high risk (t(6;9), 3q abnormality and del 5q).

We next assessed the expression levels of the genes within the BET-dependent program in a large series of untreated AML patients at diagnosis. Of the 26 genes within the program, we were able to assess the levels of expression of 18 of these in a large series of 436 patients.^18^ Using unsupervised clustering, this gene set classified the patient samples into six groups (Figure 3a). Moreover, these groups demonstrated statistically significant differences in the proportions of known prognostic factors such as karyotype (*P*<0.0001, Figure 3b) and mutation status for the NPM1c (*P*=0.019, Figure 3c) and FLT3-ITD mutations (*P*=0.02, Supplementary Figure 3). Of particular interest, patients with predicted sensitive genotypes, such as those patients with MLL-fusion leukemias significantly clustered within specific groups (Figure 3b). Taken together with the downregulation of this core program upon experimental treatment with I-BET, these data raise the possibility that the genes within the core program may not only provide putative transcriptional biomarkers of response, but may also assist with determining sensitive patients before therapy.

### The NPM1c mutation relieves inhibition of BET proteins, facilitating upregulation of the ‘core' AML transcriptional program

Our results suggest that BET inhibition is likely to be effective in a broad range of AML subtypes. However, the molecular mechanism underpinning this efficacy is likely to vary depending on the mutational spectrum of individual cases, with BET proteins serving as a common terminal effector of transcription downstream of these mutations. We had previously described the underlying molecular mechanism for BET inhibitors in MLL-fusion AML, and we next chose to address the mechanism of action of this emerging epigenetic therapy in NPM1c AML. Our previous characterization of the nuclear BET protein interactome had identified an interaction between BRD4 and a proportion of WT NPM1 with three separate proteomic methodologies in HL60 cells.^6^ We could further document that a portion of BRD4 colocalizes with WT NPM1 in primary samples from AML patients (Supplementary Figure 4). Although NPM1c has previously been shown to result in an excess of cytoplasmic NPM1,^19^ we noted that the vast majority of BRD4 in OCI-AML3 was retained in the nucleus (Supplementary Figure 5A). We therefore hypothesized that the NPM1–BRD4 interaction may repress the transcriptional activity of the proportion of BRD4 that interacts with NPM1. We therefore reasoned that cytoplasmic dislocation of NPM1 may abrogate this repressive interaction leading to aberrant gene expression.

**Figure 4:**
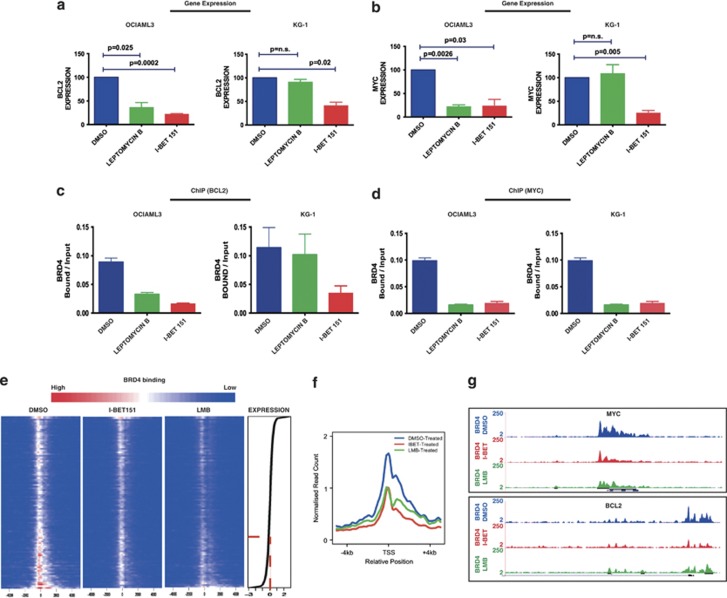
Nuclear relocalization of NPM1c phenocopies treatment with I-BET151: treatment with LMB reduces the expression of (**a**) *BCL2* and (**b**) *MYC* in OCI-AML3 but not KG-1. In contrast, I-BET151 reduces the expression of these genes in both cell lines. The gene expression changes shown were performed by real-time PCR (RT-PCR) on cDNA prepared from independent biological replicates. The expression level of target genes in the presence of DMSO was assigned a value of 100 following normalization to the B2-microglobulin (B2M) house-keeping gene whose expression in all cell lines is unaltered by I-BET151 or LMB treatment. The fold-change following treatment with I-BET151 or LMB for 6 h is shown (after normalization to the B2M house-keeping gene). Chromatin prepared from OCI-AML3 cells after 6 h of treatment with DMSO, LMB or I-BET151 was used in chromatin immunoprecipitation (ChIP) assays, followed by real-time PCR analysis. In comparison with DMSO, LMB reduces the chromatin binding of BRD4 at the transcriptional start site (TSS) of (**c**) *BCL2* and (**d**) *MYC* in OCI-AML3 but not KG-1. In contrast, I-BET151 reduces BRD4 binding at both these target genes in both cell lines. Bar graphs are represented as the mean enrichment relative to input and error bars reflect s.d. of results derived from biological triplicate experiments. (**e**) Density of BRD4 ChIP-seq reads in OCI-AML3 shown as heat maps centered on the TSS of annotated genes with 5 kb of flanking sequence either side. Heat maps are shown for BRD4 binding following treatment with DMSO, I-BET151 and LMB. Red color indicates higher density of reads. The decrease in BRD4 binding occurs primarily over genes that show a significant decrease in expression following treatment with I-BET151 (red dotted line). (**f**) Mean enrichment pattern for BRD4 binding was profiled across all annotated TSSs following treatment of OCI-AML3 with DMSO, I-BET151 and LMB. These data demonstrate that similar to treatment with I-BET151, the relocation of NPM1c with LMB reduces BRD4 binding at chromatin. (**g**) The decrease in BRD4 binding by LMB and I-BET151 is demonstrated across the *BCL2* and *MYC* loci.

Normal cytoplasmic shuttling of NPM1 occurs in a CRM1-dependent manner, and to test our hypothesis we restored nuclear NPM1 with the CRM1 inhibitor Leptomycin B (LMB).^11^ The prediction of our hypothesis would be that LMB treatment would restore nuclear NPM1c and negatively regulate BRD4-dependent transcription at critical loci. As LMB treatment may lead to pleiotrophic effects we first established that LMB treatment for a period of 6 h did not lead to discernible phenotypic effects on apoptosis and cell cycle progression in the cell lines (Supplementary Figure 5B and C and data not shown). We then tested the effects of LMB treatment on gene expression and BRD4 binding at two critical loci from the core transcriptional program, *BCL2* and *C-MYC*, in *NPM1c* mutant OCI-AML3 cells. As a further control we also treated the I-BET sensitive but *NPM1* WT KG-1 cells with LMB. For comparison, the effects on transcription and BRD4 binding were also assessed in both cell lines following I-BET treatment. In OCI-AML3 (*NPM1c*) cells, relocation of NPM1c into the nucleus with LMB phenocopied treatment with I-BET in downregulating expression of both *BCL2* and *C-MYC* (Figures 4a and b). In contrast, in *NPM1* WT KG-1, no effect was seen following LMB treatment and only I-BET treatment decreased expression of *BCL2* and *C-MYC* (Figures 4a and b). In OCI-AML3, as anticipated, we found that the decrease in gene expression of *BCL2* and *C-MYC* following LMB and I-BET treatment was accompanied by a concomitant decrease in BRD4 binding at the transcriptional start sites of these genes (Figures 4c and d). However, in KG-1 cells, BRD4 binding only decreased following I-BET treatment (Figures 4c and d). To assess if this relationship was observed at a global level we performed chromatin immunoprecipitation followed by next-generation sequencing (ChIP-seq) experiments for BRD4 binding following treatment with dimethyl sulfoxide vehicle, I-BET or LMB in OCI-AML3 cells. In keeping with the expression patterns following these perturbations, the binding of BRD4 was only marginally altered at the majority of loci whose expression remained unchanged following treatment with either LMB or I-BET (Figure 4e). However, in genes whose expression was significantly downregulated following I-BET administration, an obvious decrease in BRD4 binding was demonstrated at the transcriptional start site following treatment with either I-BET or LMB (Figures 4e–g).

**Figure 5:**
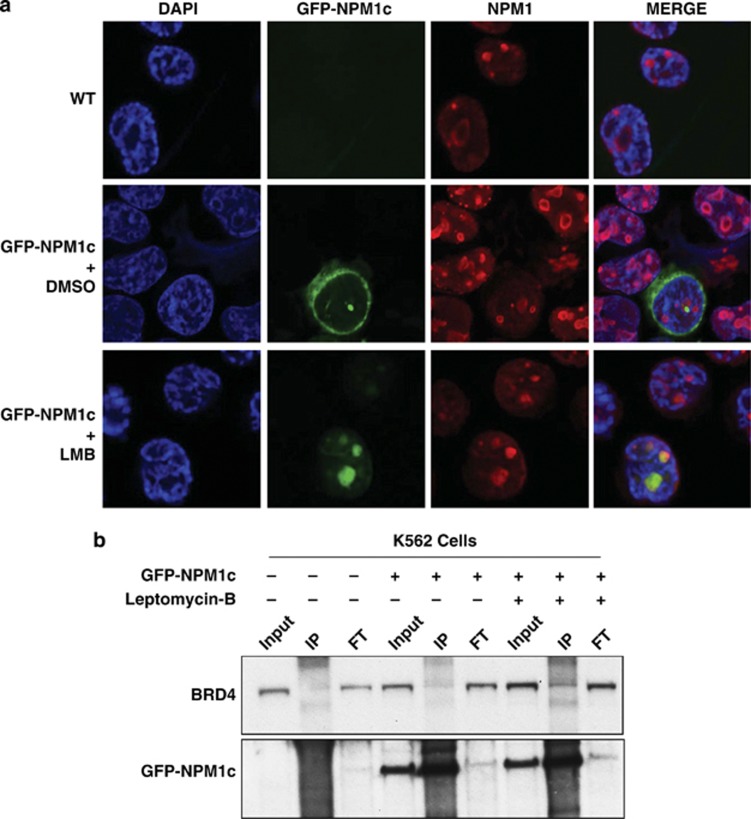
Relocation of NPM1c into the nucleus leads to a re-association with BRD4: in a cell line diploid for wild-type NPM1 we trasfected mutant NPM1 N-terminally tagged with green fluorescent protein (GFP-NPM1c). Transfection efficiency was between 10–20%. We were able to distinguish wild-type NPM1 from mutant NPM1 with an antibody raised against amino acids 1–100 of NPM1 and therefore does not recognize GFP-NPM1c. In this isogenic cellular background (**a**) confocal immunofluorescence microscopy images show that the subcellular localization of NPM1c is within the cytoplasm. However, following treatment with LMB, NPM1c is relocated back into the nucleus/nucleolus. (**b**) From the subset (10–20%) of cells expressing GFP-NPM1c, we demonstrate that the relocalization of NPM1c into the nucleus/nucleolus leads to an increased association with BRD4. Also demonstrated is 5% input and 5% of the flow through (FT) fraction following immunoprecipitaion (IP).

To expand on these data, we used K562 cells (which carry germline *NPM1* alleles) and expressed GFP-NPM1c in a proportion of these cells, allowing us to mechanistically test our hypothesis in an isogenic background (Supplementary Figure 6). In these cells we could demonstrate that LMB relocated the majority of the NPM1c to the nucleus (Figure 5a) and this restored the interaction between BRD4 and NPM1c (Figure 5b). Furthermore, in these cells the expression of NPM1c increased the expression of exemplar genes from our core transcriptional program (Supplementary Figure 6), in comparison with control cells. Taken all together, these data are consistent with our hypothesis that nuclear NPM1 exerts a repressive effect on BRD4 and decreases transcription at certain critical loci via a reduction of BRD4 chromatin binding. Loss of this inhibitory effect via NPM1c mutation would explain, at least in part, the aberrant transcriptional regulation evident in NPM1c AML and would also explain the sensitivity of this disease to BET inhibition.

### I-BET is a promising preclinical agent against NPM1c AML

**Figure 6:**
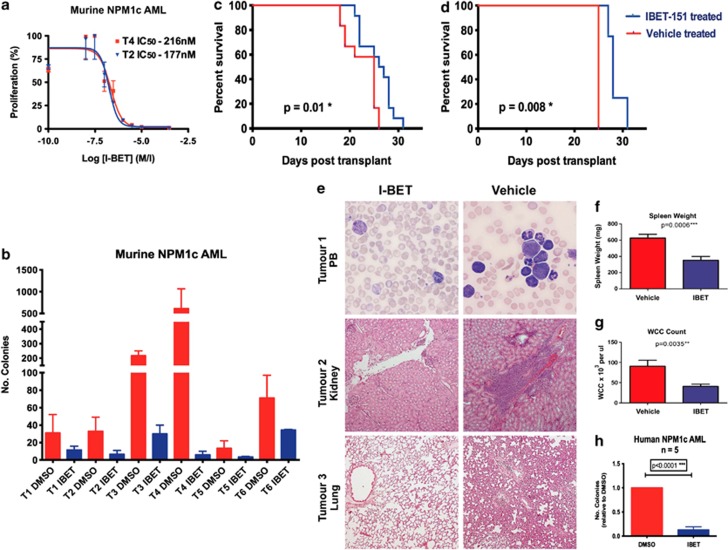
I-BET151 is efficacious *in vitro* and *in vivo* in a murine model of NPM1c AML and primary human NPM1c AML samples. Six different murine NPM1c AML were tested in (**a**) cell proliferation and (**b**) clonogenic assays. These data demonstrate that I-BET151 is effective *in vitro* in multiple NPM1c AML cases that carry a variety of other collaborating mutations. (**c**) Kaplan–Meier curve demonstrating that treatment of NOD-SCID mice transplanted with 1 × 10^7^ murine NPM1c leukemic cells show a significant increase in overall survival following treatment with I-BET151 at the experimental end point. Here, 24 mice were split into three equal groups and transplanted with three different NPM1c AML. Half of each group were treated with vehicle and half treated with I-BET151. Treatment was commenced on day 10 post transplantation. (**d**) Kaplan–Meier curve from the subgroup of mice that received the NPM1c AML, which contained a concurrent gain of function mutation in FLT3. These data show a significant increase in overall survival following treatment with I-BET151 at the experimental end point. (**e**) Top panel—Romanowsky stain of a peripheral blood smear from a vehicle- and I-BET151-treated mouse showing the morphological appearance of the increased circulating leukemic cells in the control mice. Middle panel—Haematoxylin and eosin stained histological sections of the renal parenchyma and lung (lower panel) of control and treated mice. These data demonstrate overt extramedullary leukemic infiltration of the kidney and lung in the control mouse. In contrast, a relatively normal architecture is seen in the treated animal. (**f**) Spleen weights and (**g**) total circulating white cell count (WCC) from all the vehicle and treated mice at the time of necropsy. (**h**) Clonogenic assays with 5 × 10^3^–1 × 10^4^ primary human NPM1c AML cells from five different patients. Cells were plated in cytokine-supplemented methylcellulose in the presence of vehicle (DMSO) or I-BET151. These show a marked reduction of colony formation in the presence of I-BET151.

NPM1c AML represents one of the largest subtypes of AML patients and has a variable prognosis, dependent upon the presence of cooperating mutations.^20^ For example, co-occurrence of NPM1c with internal tandem duplication of the FLT3 gene (*FLT3-ITD*) and DNMT3A mutations (as occurs in the OCI-AML3 cell line) is common and confers a relatively poor prognosis.^20, 21^ Therefore, to further assess the efficacy of I-BET against NPM1c AML in cooperation with a number of other mutations, we utilized leukemia cells from an elegant, recently described murine model. In this model, NPM1c drives leukemia in cooperation with a range of other mutations generated through transposon-based insertional mutagenesis leading to unique tumors in each case with identified cooperating mutations in addition to NPM1c (Vassiliou *et al.*^15^). Leukemia cells from all six independent tumors tested demonstrated sensitivity to I-BET in both liquid culture (with IC_50_ values ranging from 120–312 nM) and colony formation assays (Figures 6a and b). Furthermore, sensitivity of NPM1c AML to I-BET *in vivo* was confirmed following transplant of leukemia cells from three separate NPM1c AML tumors into NOD-SCID IL-2Rγ −/− recipient mice. In these preclinical models, I-BET therapy was not instituted until overwhelming disease burden was demonstrated by flow cytometry of peripheral blood (day 11 post transplant), mimicking the presentation of patients with AML in the clinic. Across all tumors, a significant survival advantage (*P*=0.01) was demonstrated for mice treated with I-BET in comparison with vehicle-treated controls (Figure 6c and Supplementary Tables 3 and 4). Of specific relevance for human AML, in leukemia driven by both NPM1c and a FLT3 activating mutation a highly significant survival advantage was demonstrated (*P*=0.008)(Figure 6d). Although the survival advantage observed in this *in vivo* model is modest, it is important to note that in contrast to many published studies assessing the efficacy of small molecules *in vivo*, we commenced treatment only once established disease was demonstrated to mimic the scenario observed clinically. Despite this, we still established a significant survival advantage in keeping with those previously reported with BET inhibitors and other novel epigenetic therapies in models of AML,^7, 22^ lymphoma^9^ and multiple myeloma.^8^ Moreover, at necropsy, tumor bulk was considerably lower in all recipient mice treated with I-BET, as evidenced by histology, peripheral white cell count and spleen weight (Figures 6e–g).

Finally, we tested the efficacy of I-BET to inhibit clonal growth of samples from NPM1c AML patients. The majority (four out of five) of these samples also carried FLT3-ITD mutations (Supplementary Table 1). As is shown, colony number was significantly decreased in all cases and increased apoptosis and characteristic transcriptional changes were also evident (Figure 6h, Figure 1e and Figure 2c). Together with the murine studies, these data demonstrate that, regardless of the nature of the cooperating mutation, NPM1c leukemias are highly sensitive to growth inhibition by I-BET.

## Discussion

AML remains an unmet medical need. Novel therapies are therefore urgently required, particularly therapeutics with toxicities acceptable for the increasingly elderly population who present with AML. In this report, we demonstrate that a wide range of disparate AML subtypes are sensitive to growth inhibition *in vitro* following I-BET151 treatment, a specific inhibitor of BET proteins that we have previously shown to be of low toxicity in preclinical studies.^6, 23^ These data suggest the clinical utility of BET inhibition across a number of AML subtypes. Of note, the related BET inhibitor I-BET762^24^ is currently in clinical trials for NUT midline carcinoma, an aggressive epithelial carcinoma characterized by genomic rearrangement of BRD4 or BRD3 (ClinicalTrials.gov identifier: NCT01587703). Our findings with I-BET151 and those with another inhibitor of BET proteins, JQ1, in AML^6, 7^ now provide the basis for early phase clinical trials in these often fatal malignancies.

Our findings also demonstrate that BET proteins are transcriptional regulators of genes critically required for leukemogenesis. We have previously defined a set of genes in MLL-rearranged AML cell lines whose expression is altered following treatment with I-BET, but it was unknown whether a similar transcriptional program was required in other sensitive AML subtypes. When similar experiments were performed in other AML cell lines, an obvious overlap with the MLL program was evident. This overlap contains 26 genes that are consistently downregulated and includes the critical regulators of myelopoiesis and leukemogenesis *BCL2, C-MYC* and *IRF8*. Although MLL fusion and NPM1c AML subtypes are associated with the aberrant expression of *HOXA* genes,^16, 25^ the common transcription program and the profound *in vitro* and *in vivo* effects of BET inhibitors in these leukemias are independent of downregulation of the *HOXA* genes. Interestingly, a number of the common genes derived from our analyses in AML cell lines contain super-enhancers that are exquisitely sensitive to BRD4 inhibition by I-BET151. Notably, many of the genes downregulated in the human AML cell lines were also downregulated following I-BET treatment in multiple primary AML samples from patients with disparate genotypes. In addition, our 26-gene signature could classify AML into distinct molecular subgroups raising the possibility that this core transcriptional signature can provide biomarkers to predict sensitivity to BET inhibition and may also be used prospectively to monitor patient response to BET inhibition in real time. We propose that this gene set comprises a ‘core' BET-responsive transcriptional program abnormally regulated in AML, and that abrogation of this program underpins the apoptosis and cell cycle arrest that are uniform upon I-BET therapy.

**Figure 7:**
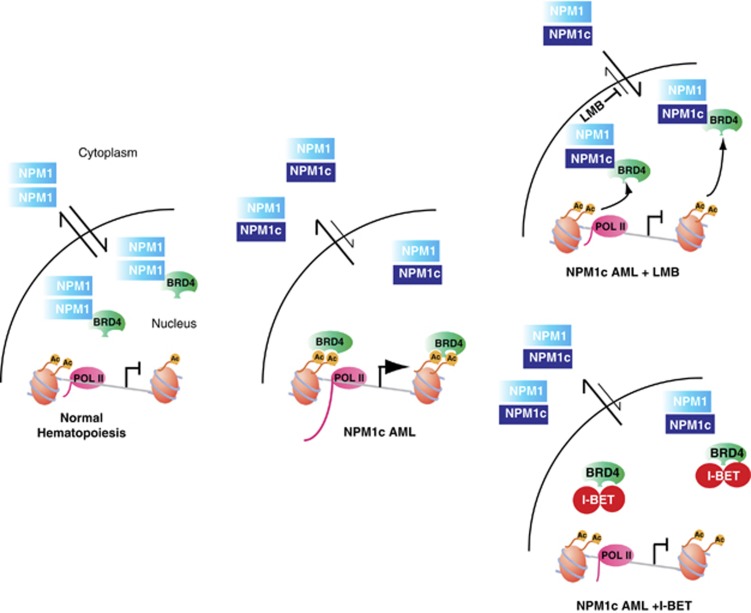
Model for the molecular mechanism of action for I-BET in NPM1c AML. Wild-type nucleophosmin 1 (NPM1) associates with a small nuclear pool of BRD4 (left panel) and exerts an inhibitory effect on its transcriptional activity. The NPM1c mutation in AML alters this equilibrium (middle panel) as a significant proportion of NPM1 is dislocated into the cytoplasm without BRD4, which is then free to drive the transcription of its target genes. I-BET displaces the binding of BRD4 from chromatin (right upper panel) leading to the repression of the target genes and relocation of NPM1c into the nucleus with LMB phenocopies I-BET (left lower panel), as it leads to a re-association of NPM1c with BRD4 off the chromatin template also resulting in transcriptional repression.

Our studies also shed direct light on one of the prevailing mysteries in AML biology: the mechanisms of transcriptional dysregulation in NPM1c AML. We have previously demonstrated that WT NPM1 and BRD4 interact and further demonstrate this interaction in primary AML cells with WT NPM1. Our data predict that NPM1c mutations relieve an inhibitory interaction with BRD4 through cytosolic dislocation of NPM1c and WT NPM1, allowing BRD4 to activate aberrant transcription in NPM1c AML (Figure 7). In support of our hypothesis, we demonstrate that restoration of nuclear NPM1c following treatment with the CRM1 inhibitor LMB restores the NPM1–BRD4 interaction and downregulates expression of critical genes, such as *BCL2* and *C-MYC* through a reduction in BRD4 recruitment to chromatin. Restoration of nuclear NPM1 following LMB treatment closely phenocopies the inhibitory effects of I-BET on transcription and BRD4 chromatin binding (Figure 7).

We and others have previously demonstrated the preclinical efficacy of BET inhibition in MLL-rearranged leukemias, and here we report a similar *in vitro* sensitivity of AML with NPM1c mutations. NPM1c AML represents one of the single largest AML subgroups, comprising around 35% of all cases, and has a variable prognosis. This prognosis is largely dependent upon the presence or absence of other ‘cooperating' mutations, particularly the FLT3-ITD mutation. Importantly, our findings demonstrate that NPM1c AML cells are uniformly sensitive to I-BET inhibition regardless of the nature of the cooperating mutations (Figures 6a–h and Supplementary Tables 1, 3 and 4). These include mutations known to predict poor prognosis, including DNMT3A and the FLT3-ITD.^20^ Of particular clinical relevance, around 15% of cases of AML harbor both an NPM1c mutation and a FLT3-ITD, and these patients have a relatively poor prognosis.^20^ Here, we demonstrate sensitivity to I-BET in both primary human AML cells and a mouse model that carry both an NPM1c mutation and an activating mutation of FLT3, suggesting significant clinical utility in this poor-risk subgroup.

Taken together, our data greatly inform the pathogenesis of NPM1c AML and provide compelling evidence supporting clinical trials of BET inhibitors across multiple AML subtypes. Moreover, our data identify potential predictive biomarkers of sensitivity and response to inform these studies. Novel, nontoxic, targeted therapies are desperately required in AML, and we eagerly await results of trials of BET inhibition in this aggressive hematological malignancy.

## Notes

DL, PJ, GD and RKP are employees of Glaxo Smith Kline. KL is an employee of Pfizer. The remaining authors declare no conflict of interest.
