# Supplementary material for: Recurrent mutations, including NPM1c, activate a BRD4-dependent core transcriptional program in acute myeloid leukemia
Source: Leukemia. 2013 Dec 13;28(2):311–20. doi: 10.1038/leu.2013.338 (PMC3918873; doi:10.1038/leu.2013.338)

Supplementary Figure 1

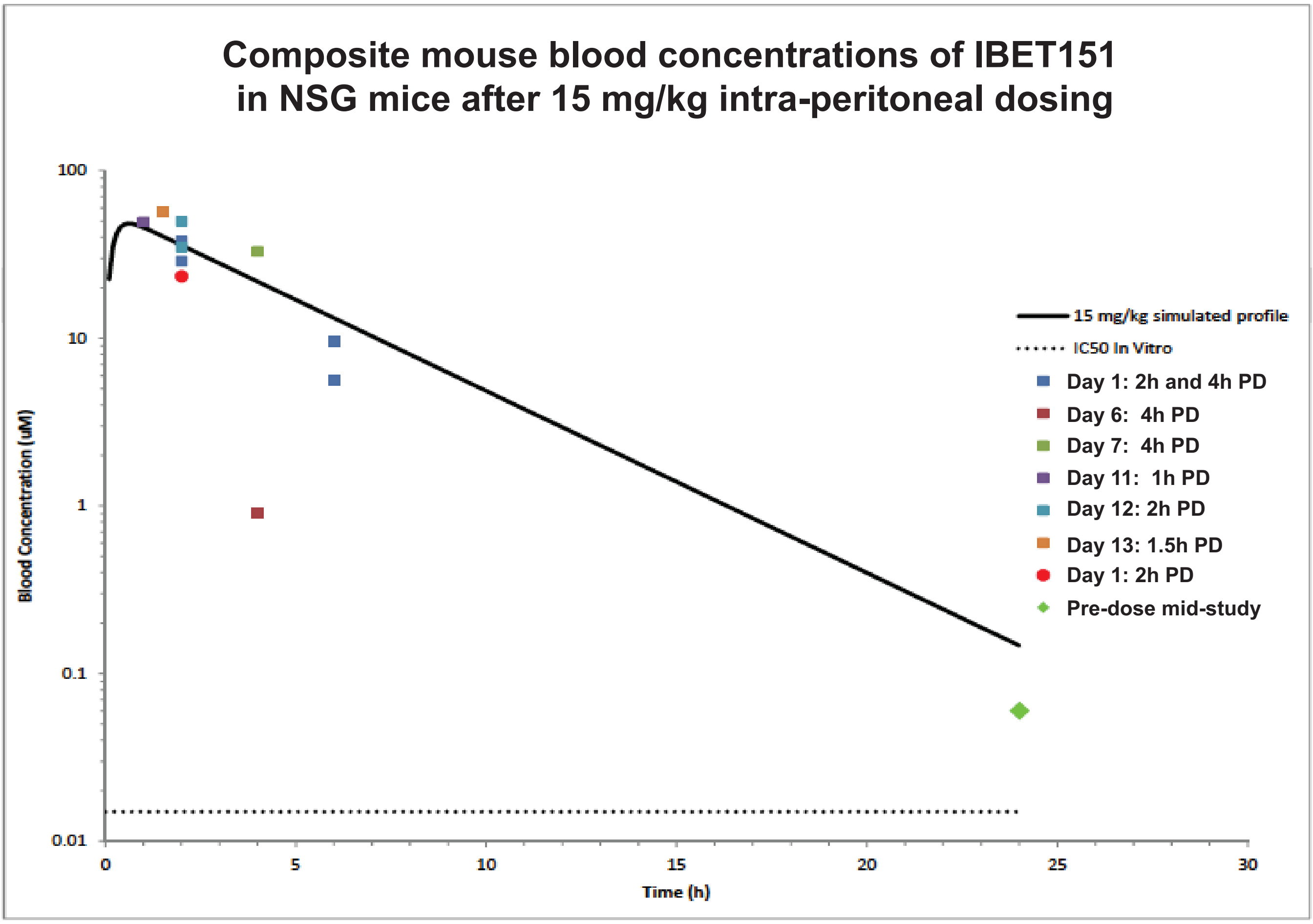

Supplementary Figure 2

A

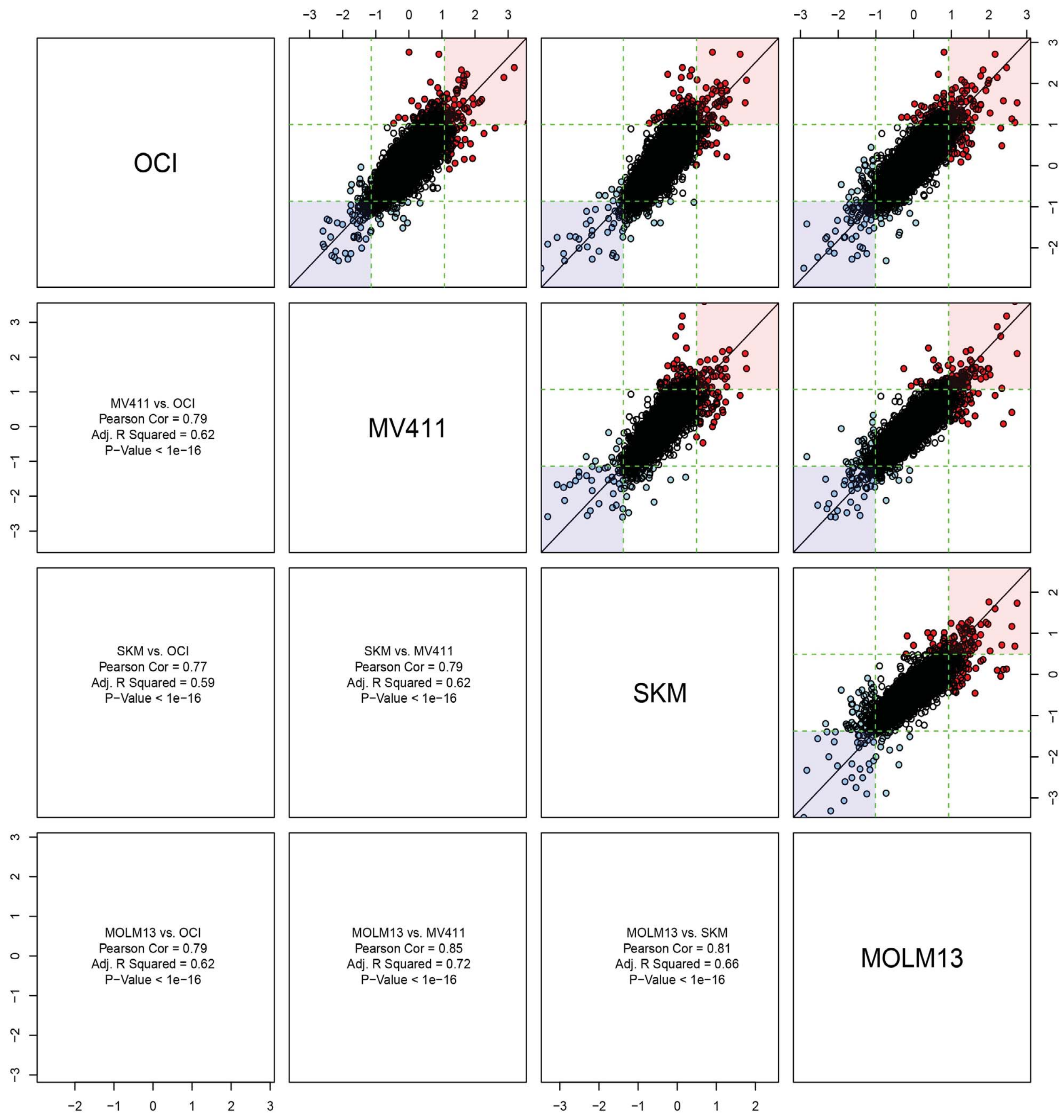

B

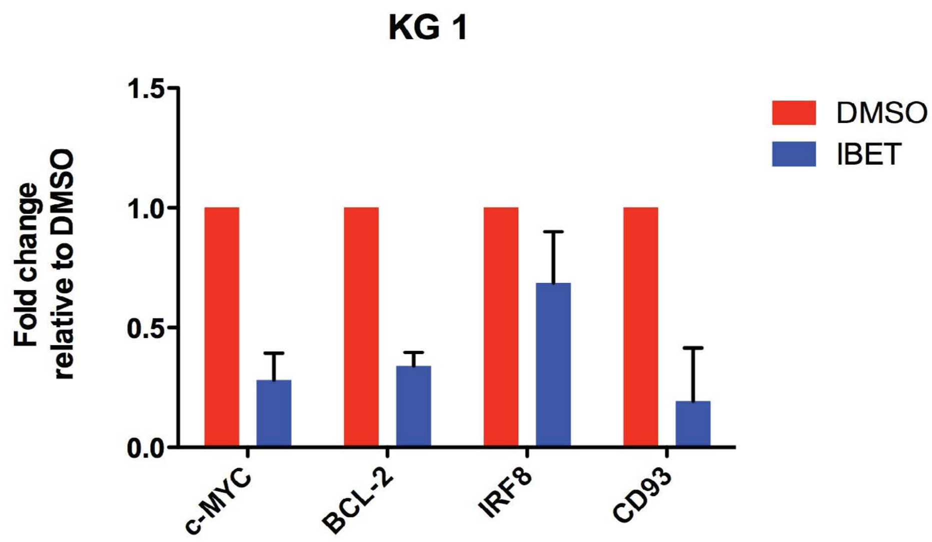

Supplementary Figure 3

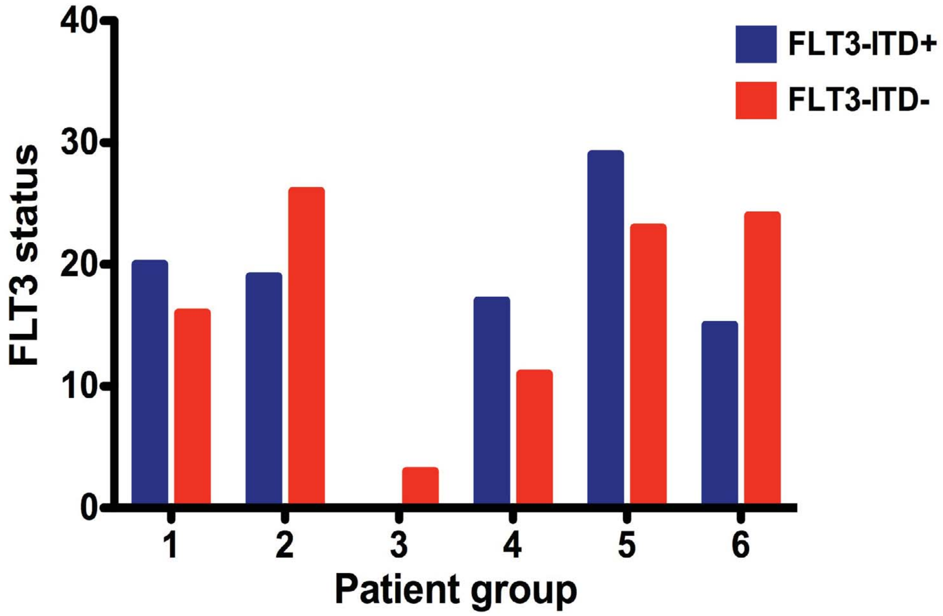

Supplementary Figure 4

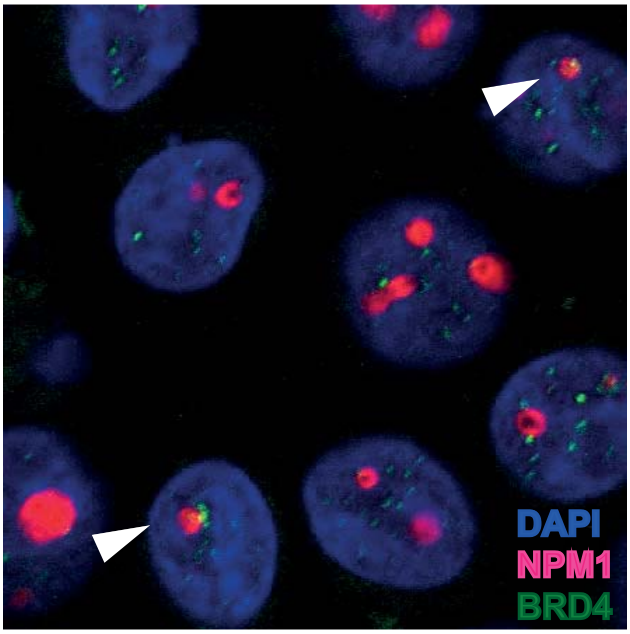

Supplementary Figure 5

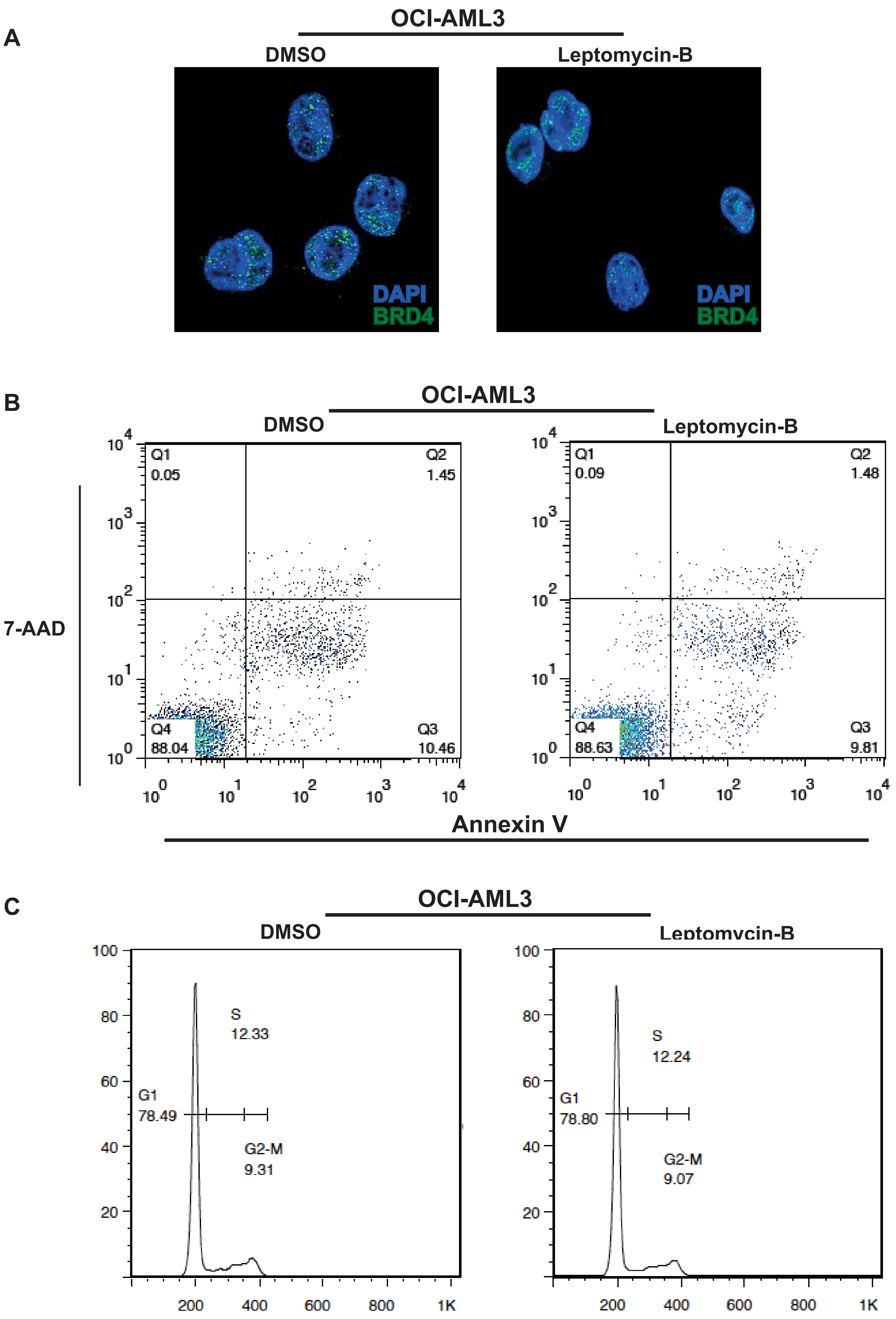

Supplementary Figure 6

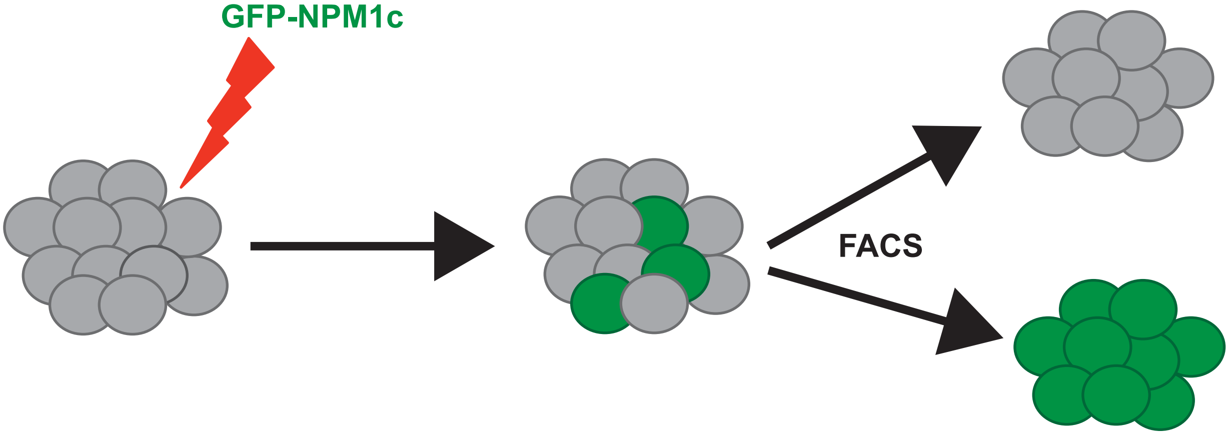

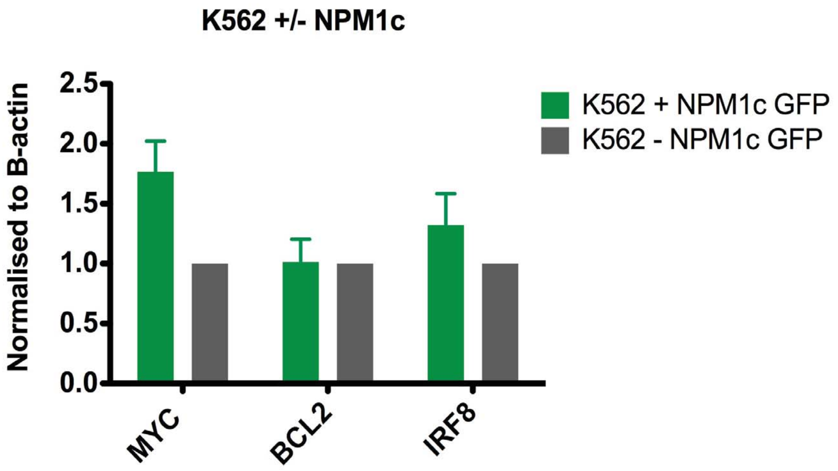

Supplement: Supplementary Figures [file leu2013338x1.pdf]
