# Supplementary material for: Recurrent mutations, including NPM1c, activate a BRD4-dependent core transcriptional program in acute myeloid leukemia
Source: Leukemia. 2013 Dec 13;28(2):311–20. doi: 10.1038/leu.2013.338 (PMC3918873; doi:10.1038/leu.2013.338)
Supplement: Supplementary Tables [file leu2013338x2.pdf]

Patient	Sex	Age	Karyotype	NPM1 status	FLT3 status	Fig 1 plating	Fig 1 Annexin	Fig 2 Gene Exp	Fig 4 plating
**PT1**	M	80	Normal	WT	WT	N	Y	Y	N
**PT-2**	M	50	t(15;17)	WT	WT	N	N	Y	N
**PT-3**	M	47	Normal	NPM1c	TKD	N	Y	Y	N
**PT-4**	F	79	i17(q)	WT	WT	N	Y	Y	N
**PT-5**	M	15	Normal	WT	WT	N	N	Y	N
**PT-6**	M	70	Normal	NPM1c	WT	N	Y	Y	Y
**PT-7**	F	71	Normal	WT	WT	Y	N	N	N
**PT-8**	M	79	Ab Ch3(q26?) +marker	WT	ITD	Y	N	N	N
**PT-9**	M	50	t(15;17)	WT	ITD	Y	N	N	N
**PT-10**	M	56	t(3;7)	WT	WT	Y	N	N	N
**PT-11**	F	63	N/A	WT	ITD	Y	N	N	N
**PT-12**	M	71	Normal	N/A	WT	Y	N	N	N
**PT-13**	F	68	Add 11(p15)	NPM1c	WT	N	Y	N	N
**PT-14**	M		Normal	NPM1c	ITD	N	N	N	Y
**PT-15**	F	56	Normal	NPM1c	ITD	N	N	N	Y
**PT-16**	M	47	Normal	NPM1c	ITD	N	N	N	Y
**PT- 17**	F	80	U	NPM1c	ITD	N	N	N	Y

Supplementary Table 1. Details of the seventeen primary human AML samples used in this study

Supplementary Table 2

Genes within the core signature

ANKRD41
BCL2
C12orf24
CACNA2D3
CCDC86
CSF1R
FAM136A
FLJ39827
IRF8
LAT2
LOC728492
LRRC33
MLKL
MS4A6A
MYBPH
MYC
NOP16,HSPC111
PHF15
PLD6
RRS1
SLA
SLC2A5
TIGD5
TNS3
TRIP6
TSEN2

Date of transplant	Unique ID	Tumour	Date/type of treatment	Date death	Survival post Transp (days)	Survival post Rx (days)	Reason for culling
05/04/12	196661 1L	Tumour 1	16/04/12 I-BET	30/04/12	25	14	Weight loss > 20%
05/04/12	196661 1R	Tumour 1	16/04/12 I-BET	01/05/12	26	15	Weight loss > 20%
05/04/12	196661 NM	Tumour 1	16/04/12 I-BET	04/05/12	29	18	Weight loss > 20%
05/04/12	196661 1L1R	Tumour 1	16/04/12 I-BET	03/05/12	28	17	Weight loss > 20%
05/04/12	197887 2L	Tumour 1	16/04/12 Vehicle	26/04/12	21	10	Hind limb paralysis
05/04/12	197887 2R	Tumour 1	16/04/12 Vehicle	01/05/12	26	15	Weight loss > 20%
05/04/12	197887 1L	Tumour 1	16/04/12 Vehicle	30/04/12	25	14	Weight loss > 20%
05/04/12	197887 1R	Tumour 1	16/04/12 Vehicle	01/05/12	26	15	Hind limb paralysis
05/04/12	196660 2L	Tumour 2	16/04/12 I-BET	27/04/12	22	11	Weight loss > 20%
05/04/12	196660 2R	Tumour 2	16/04/12 I-BET	27/04/12	22	11	Weight loss > 20%
05/04/12	196660 NM	Tumour 2	16/04/12 I-BET	26/04/12	21	10	Weight loss > 20%
05/04/12	196660 1L1R	Tumour 2	I16/04/12 -BET	27/04/12	22	11	Weight loss > 20%
05/04/12	196662 2L	Tumour 2	16/04/12 Vehicle	23/04/12	18	7	Weight loss > 20%
05/04/12	196662 2R	Tumour 2	16/04/12 Vehicle	24/04/12	19	8	Weight loss > 20%
05/04/12	196662 NM	Tumour 2	16/04/12 Vehicle	24/04/12	19	8	Weight loss > 20%
05/04/12	196662 1L1R	Tumour 2	16/04/12 Vehicle	23/04/12	18	7	Weight loss > 20%
05/04/12	196663 1L	Tumour 3	16/04/12 I-BET	02/05/12	27	16	Weight loss > 20%
05/04/12	196663 1R	Tumour 3	16/04/12 I-BET	03/05/12	28	17	Weight loss > 20%
05/04/12	196663 NM	Tumour 3	16/04/12 I-BET	03/05/12	28	17	Weight loss > 20%
05/04/12	196663 1L1R	Tumour 3	I16/04/12 -BET	06/05/12	31	20	Weight loss > 20%
05/04/12	197888 1L	Tumour 3	16/04/12 Vehicle	30/04/12	25	14	Hind limb paralysis
05/04/12	197888 1R	Tumour 3	16/04/12 Vehicle	30/04/12	25	14	Weight loss > 20%
05/04/12	197888 2L	Tumour 3	16/04/12 Vehicle	30/04/12	25	14	Hind limb paralysis
05/04/12	197888 2R	Tumour 3	16/04/12 Vehicle	30/04/12	25	14	Weight loss > 20%

Supplementary Table 3. Life tables for transplanted mice. Treated mice are highlighted in blue, non-treated vehicle controls have no highlighting**.**

**NPM1c murine tumour**	**Common integration sites**
**Tumour 1**	E2f8<br>RP23-106P7.2<br>Aldh1l1<br>Ch11:54083069: intergenic<br>Ch8:83417895:intergenic
**Tumour 2**	Iqgap2<br>CSF2<br>Ch7:152239044:intergenic<br>Ch16:42681266: intergenic
**Tumour 3**	Flt3<br>Ptpn1<br>Cux1<br>Pilcam<br>Tnks<br>Nf1

Supplementary Table 4. Cooperating mutations in separate murine NPM1c murine models
