# Supplementary material for: Recurrent mutations, including NPM1c, activate a BRD4-dependent core transcriptional program in acute myeloid leukemia
Source: Leukemia. 2013 Dec 13;28(2):311–20. doi: 10.1038/leu.2013.338 (PMC3918873; doi:10.1038/leu.2013.338)
Supplement: Supplementary Legends [file leu2013338x3.doc]

**Supplemental Information:**

**Supplementary Figure Legends**

**Supplementary Figure 1:** Following initiation of treatment in the NOD-SCID IL-2Rγ -/- recipient mice, single individual blood samples (n = 4 per time point) were obtained at 1, 6, and 24 h post I-BET151 dose (first dose) to confirm system exposures at the start of the study. Additional samples were taken as required. Blood samples were treated and analyzed as described previously (Dawson et al., 2011).

**Supplementary Figure 2:** (A) Correlation plots between the gene expression changes in the OCI-AML3, SKM1, MOLM13 and MV411 cell lines. The top panels of the grid show the correlation between the intersecting cell lines, whilst the lower panels give the statistics for the correlation (Pearson correlation, and adjusted R-squared and p-value for a fitted linear model). Genes showing significant differential expression (p <= 0.01; fold change >= 2-fold) are highlighted in red (up-regulated) or blue (down-regulated). (B) In KG1 cells, the expression levels of some of the core target genes were assessed by real-time PCR (RT-PCR) on cDNA prepared from independent biological replicates. The expression level in the presence of DMSO was assigned a value of 100 following normalization to the B2-microglobulin house keeping gene whose expression in all cell lines is unaltered by DMSO or I-BET treatment.

**Supplementary Figure 3:** Eighteen of twenty-seven genes (80%) from the core transcription signature were differentially expressed across a cohort of 436 AML patients as shown in Figure 3. The gene set could classify this cohort into 6 groups through the use of unsupervised clustering. Significant differences in FLT3-ITD status were also noted (P = 0.02).

**Supplementary Figure 4:** Confocal immunofluoresence microscopy images of primary AML patient samples with wildtype NPM1 showing co-localisation of a small fraction of NPM1 (red) with BRD4 (green).

**Supplementary Figure 5:** (A) Confocal immunofluorescent microscopy images in OCI-AML3 demonstrating that BRD4 (green) is primarily nuclear and this subcellular localization is not altered following treatment with leptomycin-B. The nuclei of OCI-AML3 are stained blue with 4,6-diamidino-2-phenylindole (DAPI). OCI-AML3 cells were treated for six hours with either DMSO (vehicle) or Leptomycin B (LMB). (B) The degree of apoptosis in OCIAML3 was assessed using the vital dye 7-amino-actinomycin D (7-AAD) and Annexin V in cells following 72 hours incubation with DMSO or LMB. These data demonstrate no induction of apoptosis at this time point. (C) Cell cycle progression in OCI-AML3 was assessed 24 hours after incubation with DMSO or LMB. These data also demonstrate no change in cell cycle progression.

**Supplementary Figure 6:**

K562 cells (which carry germline *NPM1* alleles) were transfected with GFP-NPM1c. A proportion of these cells (between 15-20%) expressed GFP-NPM1c. GFP positive cells were sorted from GFP negative cells thus allowing us to test the expression of BRD4 target genes in the presence or absence of NPM1c in an isogenic background.

**Supplementary Table 1:**

Table containing clinical information about the seventeen primary AML samples used for various functional and molecular analyses in the manuscript.

**Supplementary Table 2:**

This excel table contains a list of all the 27 genes in OCI-AML3, SKM1, MV41;11 and MOLM13 that comprise the core transcription program alter by BET inhibitors.

**Supplementary Table 3:**

Life table for the in vivo experiments

**Supplementary Table 4:**

Table listing the cooperating mutations seen in the three murine NPM1c AML’s used in the in vivo experiment.
